# Supplementary material for: Exploration of Psychiatry Residents’ Attitudes toward Patients with Substance Use Disorder, Bipolar Disorder and Schizophrenia in Saudi Arabia
Source: Behav Sci (Basel). 2023 Aug 1;13(8):642. doi: 10.3390/bs13080642 (PMC10451806; doi:10.3390/bs13080642)
Supplement: Supplementary file 1 [file behavsci-13-00642-s001.zip › behavsci-2436710-supplementary.pdf]

Supplementary Table S1: Summary of statements and responses for the assessment of participants' attitudes toward patients with three different conditions.

Statements	Strongly disagree	Disagree	Not sure but probably disagree	Not sure but probably agree	Agree	Strongly agree
**Substance Use Disorder:**						
I prefer not to work with Substance Use Disorder patients.	9 (11.4)	23 (29.1)	18 (22.8)	14 (17.7)	12 (15.2)	3 (3.8)
Substance Use Disorder patients irritate me.	15 (19.0)	24 (30.4)	21 (26.6)	13 (16.5)	5 (6.3)	1 (1.3)
I enjoy giving extra time to Substance Use Disorder patients.	6 (7.6)	23 (29.1)	29 (36.7)	13 (16.5)	7 (8.9)	1 (1.3)
Substance Use Disorder patients are particularly difficult for me to work with.	4 (5.1)	18 (22.8)	20 (25.3)	21 (26.6)	12 (15.2)	4 (5.1)
Working with Substance Use Disorder patients is satisfying.	4 (5.1)	25 (31.6)	28 (35.4)	15 (19.0)	7 (8.9)	0 (0.0)
I feel especially compassionate toward Substance Use Disorder patients.	4 (5.1)	24 (30.4)	23 (29.1)	9 (11.4)	15 (19.0)	4 (5.1)
I wouldn't mind getting up on call nights to care for Substance Use Disorder patients.	10 (12.7)	15 (19.0)	13 (16.5)	8 (10.1)	25 (31.6)	8 (10.1)
I can usually find something that helps Substance Use Disorder patients feel better.	0 (0.0)	12 (15.2)	17 (21.5)	30 (38.0)	17 (21.5)	3 (3.8)
There is little I can do to help Substance Use Disorder patients.	3 (3.8)	24 (30.4)	16 (20.3)	18 (22.8)	16 (20.3)	2 (2.5)
Insurance plans should cover Substance Use Disorder patients to the same degree that they cover patients with other conditions.	4 (5.1)	6 (7.6)	8 (10.1)	19 (24.1)	26 (32.9)	16 (20.3)
Treating Substance Use Disorder patients is a waste of money.	40 (50.6)	24 (30.4)	10 (12.7)	3 (3.8)	1 (1.3)	1 (1.3)
**Bipolar Disorder: (.725)**						
I prefer not to work with Bipolar patients.	39 (49.4)	31 (39.2)	1 (1.3)	1 (1.3)	5 (6.3)	2 (2.5)
Bipolar patients irritate me.	34 (43.0)	33 (41.8)	8 (10.1)	4 (5.1)	4 (5.1)	0 (0.0)
I enjoy giving extra time to Bipolar patients.	1 (1.3)	11 (13.9)	12 (15.2)	14 (17.7)	30 (38.0)	11 (13.9)
Bipolar patients are particularly difficult for me to work with.	11 (13.9)	43 (54.4)	12 (15.2)	10 (12.7)	3 (0.0)	0 (0.0)
Working with Bipolar patients is satisfying.	1 (1.3)	1 (1.3)	8 (10.1)	16 (20.3)	30 (38.0)	23 (29.1)
I feel especially compassionate toward Bipolar patients.	1 (1.3)	4 (5.1)	4 (5.1)	16 (20.3)	36 (45.6)	18 (22.8)
I wouldn't mind getting up on call nights to care for Bipolar patients	0 (0.0)	2 (2.5)	2 (2.5)	9 (11.4)	47 (59.5)	19 (24.1)

I can usually find something that helps Bipolar patients feel better	0 (0.0)	2 (2.5)	6 (7.6)	16 (20.3)	40 (50.6)	15 (19.0)
There is little I can do to help Bipolar patients	23 (29.1)	33 (41.8)	6 (7.6)	7 (8.9)	7 (8.9)	3 (3.8)
Insurance plans should cover Bipolar patients to the same degree that they cover patients with other conditions	0 (0.0)	1 (1.3)	1 (1.3)	2 (2.5)	16 (20.3)	59 (74.7)
Treating Bipolar patients is a waste of money	66 (83.5)	11 (13.9)	0	1 (1.3)	1 (1.3)	0
**Schizophrenia: (.793)**						
I prefer not to work with Schizophrenia patients	31 (39.2)	31 (39.2)	7 (8.9)	1 (1.3)	8 (10.1)	1 (1.3)
Schizophrenia patients irritate me	40 (50.6)	29 (36.7)	4 (5.1)	2 (2.5)	4 (5.1)	0
I enjoy giving extra time to Schizophrenia patients	1 (1.3)	5 (6.3)	7 (8.9)	15 (19.0)	36 (45.6)	15 (19.0)
Schizophrenia patients are particularly difficult for me to work with	0	20 (25.3)	34 (43.0)	12 (15.2)	7 (8.9)	6 (7.6)
Working with Schizophrenia patients is satisfying	0	4 (5.1)	9 (11.4)	21 (26.6)	34 (43.0)	11 (13.9)
I feel especially compassionate toward Schizophrenia patients	1 (1.3)	3 (3.8)	6 (7.6)	12 (15.2)	33 (41.8)	24 (30.4)
I wouldn't mind getting up on call nights to care for Schizophrenia patients	0	2 (2.5)	8 (10.1)	23 (29.1)	35 (44.3)	11 (13.9)
I can usually find something that helps Schizophrenia patients feel better	0	2 (2.5)	8 (10.1)	23 (29.1)	35 (44.3)	11 (13.9)
There is little I can do to help Schizophrenia patients	14 (17.7)	34 (43.0)	14 (17.7)	10 (12.7)	6 (7.6)	1 (1.3)
Insurance plans should cover Schizophrenia patients to the same degree that they cover patients with other conditions	0	0	2 (2.5)	2 (2.5)	19 (24.1)	56 (70.9)
Treating Schizophrenia patients is a waste of money	60 (75.9)	17 (21.5)	0	0	1 (1.3)	1 (1.3)
